# Detrital food web contributes to aquatic ecosystem productivity and rapid salmon growth in a managed floodplain

**DOI:** 10.1371/journal.pone.0216019

**Published:** 2020-09-18

**Authors:** Carson A. Jeffres, Eric J. Holmes, Ted R. Sommer, Jacob V. E. Katz

**Affiliations:** 1 Center for Watershed Sciences–Davis, University of California, Davis, California, United States of America; 2 California Department of Water Resources–West Sacramento, West Sacramento, California, United States of America; 3 California Trout–San Francisco, San Francisco, CA, United States of America; University of Maine at Farmington, UNITED STATES

## Abstract

Similar to many large river valleys globally, the Sacramento River Valley has been extensively drained and leveed, hydrologically divorcing river channels from most floodplains. Today, the former floodplain is extensively managed for agriculture. Lack of access to inundated floodplains is recognized as a significant contributing factor in the decline of native Chinook Salmon *(*O*ncorhynchus tshawytscha)*. We observed differences in salmon growth rate, invertebrate density, and carbon source in food webs from three aquatic habitat types—leveed river channels, perennial drainage canals in the floodplain, and agricultural floodplain wetlands. Over 23 days (17 February to 11 March, 2016) food web structure and juvenile Chinook Salmon growth rates were studied within the three aquatic habitat types. Zooplankton densities on the floodplain wetland were 53x more abundant, on average, than in the river. Juvenile Chinook Salmon raised on the floodplain wetland grew at 0.92 mm/day, 5x faster than fish raised in the adjacent river habitat (0.18 mm/day). Two aquatic-ecosystem modeling methods were used to partition the sources of carbon (detrital or photosynthetic) within the different habitats. Both modeling approaches found that carbon in the floodplain wetland food web was sourced primarily from detrital sources through heterotrophic pathways, while carbon in the river was primarily photosynthetic and sourced from in situ autotrophic production. Hydrologic conditions typifying the ephemerally inundated floodplain—shallower depths, warmer water, longer water residence times and predominantly detrital carbon sources compared to deeper, colder, swifter water and a predominantly algal-based carbon source in the adjacent river channel—appear to facilitate the dramatically higher rates of food web production observed in the floodplain. These results suggest that hydrologic patterns associated with seasonal flooding facilitate river food webs to access floodplain carbon sources that contribute to highly productive heterotrophic energy pathways important to the production of fisheries resources.

## Introduction

The benefits of annual inundation of floodplains to riverine ecosystems and fish populations are well recognized in relatively unaltered tropical river systems [1, 2]. The impacts of anthropogenic interruption of natural flood pulses in temperate river systems, however, have only more recently begun to be characterized [3]. Systems where flood regimes have been highly modified tend to exhibit significant reductions in hydrologic and habitat diversity, biological production and fishery yields [4]. In Europe and North America, levees have been constructed along almost all major lowland rivers to control flooding and allow development of fertile floodplains for farms and cities [5]. In the Central Valley of California, approximately 95% of the historic floodplain wetlands have been drained or are no longer accessible to aquatic species behind ~3360 km of state and federal levees [6]. Extensive leveeing also diminishes the expression of the full range of life history strategies of native fish, thereby weakening the resilience of populations to natural and anthropogenic disturbance. This landscape-scale hydrologic divorce of river channel and floodplain has only recently been widely recognized and case studies of community responses, such as this paper, are not yet common [7].

The Sacramento Valley has a Mediterranean climate where summers are long and dry and almost all precipitation falls in winter and spring. This results in rivers with high annual and seasonal variability in flows, with flood pulses that historically inundated floodplains and other seasonally inundated off-channel river habitats occurring exclusively during the winter/spring wet season. Water temperatures on Central Valley floodplains inundated during the flood season are generally warmer due to decreases in depth as well as increases in surface area and water residence time compared to the relatively deep, cool and swift river channel [8, 9]. Floodplain habitats provide abundant food resources for grazing zooplankton, which ultimately provide food resources for fishes [8–11].

In this paper we explore how the food web in seasonal floodplain habitat responds differently than the main channel. We were interested in gaining a better understanding of how levees, canals and other drainage infrastructure systemically interrupt flood-pulse access to terrestrial carbon sources. Our hope was that this would provide insight into the impacts that widespread diking of stream channels and floodplain development has had on aquatic ecosystem productivity. More specifically, we sought insight into how the near elimination of long-duration, landscape-scale floodplain inundation—a hydrologic pattern that once facilitated transfer of allochthonous energy sources into the food webs of the Sacramento River—might effect the capacity of the aquatic ecosystem to produce zooplankton and native fish biomass?

**Fig 1:**
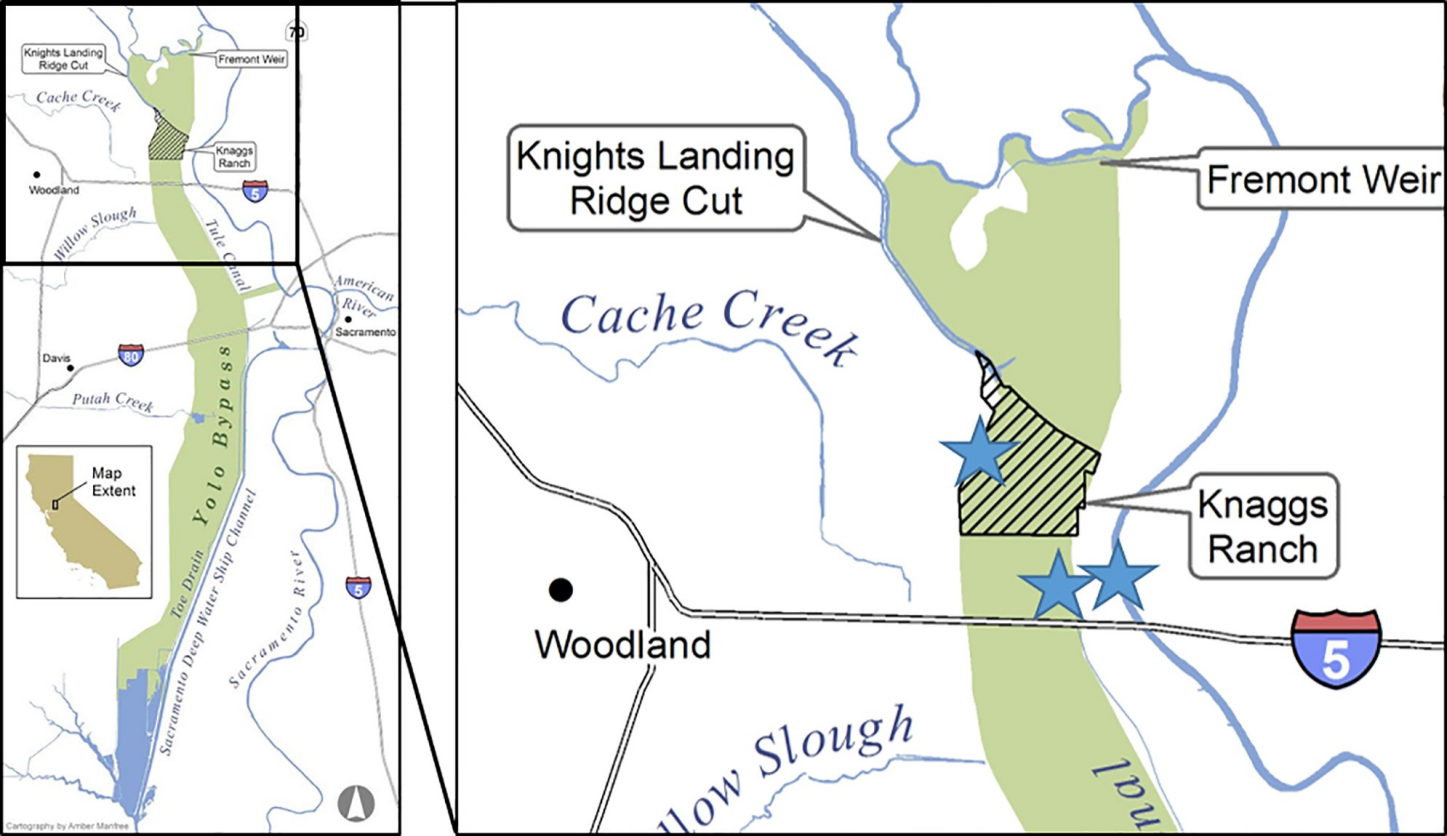
Location of study sites (blue stars) from left to right–floodplain agricultural wetlands, perennial canal, and Sacramento River.

We employed two distinct models to compare and contrast how energy (carbon) flows through aquatic food webs found in three aquatic habitat types in the Sacramento River Valley in California, USA (Fig 1) typical of those found in leveed, low gradient, alluvial valleys globally. Aquatic ecosystem metabolism is often expressed as net ecosystem productivity (NEP), which is calculated as the difference between all photosynthetic energy produced in the system (gross primary productivity, GPP) and the sum of all energy used by organisms (ecosystem respiration, ER). Autotrophic production is widely recognized as an important source of aquatic food web productivity [10] but a significant portion of the trophic energy transfer in river-floodplain systems may also move through heterotrophic food webs driven by breakdown of plant detritus [12–16]. The abundant floodplain food resources contribute significantly to the diets of juvenile salmonids accessing these off-channel habitats during flood events [17, 18] where they grow more quickly than fish confined to adjacent leveed river channels [11, 19].

This study identified differences in hydrology, carbon source and productive capacity of food webs in three aquatic habitats in the Sacramento River Valley (Fig 1) that epitomize a typical lateral cross section of a developed agricultural river valley: seasonally inundated agricultural floodplain wetlands, perennial canals engineered to drain the floodplain surface, and leveed river channels. We hypothesized that greater access to terrestrial detrital carbon sources in ephemerally inundated floodplain habitats compared to adjacent perennial drainage canal or river channel habitats should lead to aquatic food webs in these three distinct habitats characterized by:

- Higher rates of heterotrophic food web activity on the floodplain compared to the adjacent perennial canal or river channel habitats;
- Greater density of consumers on the floodplain compared to the adjacent perennial canal or river channel habitats;
- Increased growth rates of juvenile salmon reared on the floodplain habitat compared to the perennial canal or river channel habitats;
- Differing rates of ecosystem respiration (ER), gross primary production (GPP), and net ecosystem production (NEP) between riverine and floodplain habitats, with higher ER and more negative NEP on the floodplain habitat.

## Methods

### Study area

The Sacramento River is the largest river in California, draining 71,432 km^2^. Seasonal flow events from winter rains and spring snowmelt historically inundated much of the Sacramento Valley floodplain. Starting in the late 1800s levees were constructed to protect agricultural and urban development. Today, less than 5% of historical floodplain wetlands remain [20]. The exception to this hydrological disconnection of the Sacramento River from its floodplain is a series of landscape-scale flood protection projects designed to bypass flood waters around critical urban and agricultural areas. By diverting high flows onto specifically managed floodways these “bypasses” alleviate flood stress on critically important downstream levees. The largest bypass is the Yolo Bypass, a 24,000 ha floodway adjacent to the city of Sacramento and immediately upstream of the Sacramento-San Joaquin Delta (Fig 1). The Yolo Bypass is extensively farmed during summer and it floods in two out of three winters on average, although flood events are frequently shorter than a week [18]. Substantial areas of the Yolo Bypass are managed as seasonal agricultural floodplain wetlands to support wildlife, and a perennial drainage canal spans much of the eastern edge of the floodplain. This study was conducted during January through March, 2016 (with fish in enclosures from February 17 to March 11). The flooded agricultural wetland was flooded January 18, 2016, prior to placement of the fish to “initiate” aquatic food web processes, as often happens during rain events prior to natural flooding. This timing was chosen as it is the time of year when juvenile Chinook Salmon are out-migrating through the Sacramento River and would likely have access to floodplain habitats during high flow events. Moreover, during lower flow periods fish can access the perennial channels of the floodplain by migrating upstream from the lower tidal reaches of the Sacramento system [21]. Hence, wild juvenile salmon were present in both Yolo Bypass and Sacramento River during the course of our experiments.

#### Hydrology and water chemistry

Hydrology and water chemistry data were collected in order to document physical habitat differences between each of the sites, and to provide the basis for stream metabolism modeling (below). Hourly discharge and stage data in the Sacramento River and Yolo Bypass perennial drainage canal were downloaded from the California Data Exchange Center (cdec.ca.gov) at stations “VON” and “YBY”, respectively. Water residence times in the Sacramento River and the Yolo Bypass perennial drainage canal sites were calculated using a relationship between cross-sectional area and mean velocities at a variety of flows previously collected at the stations. Then that relationship was used to calculate mean water velocities from the flows that were observed during this study. The water residence time represents the mean amount of time that water spends within the cross section of the river, averaged across the whole channel, and is used as a general value for how long water stays within that section of the water body. These channel cross sections are representative of typical stream reaches of leveed rivers in the Central Valley of California. Data was collected from published values at usgs.gov for site numbers 11425500 and 11453000, respectively.

Water quality data were collected via a handheld YSI EXO (YSI Incorporated, Yellow Spring, OH, USA) sonde that measures water temperature, dissolved oxygen, salinity, pH, conductivity, and turbidity. In addition, at each location an Onset Hobo U26 dissolved oxygen and temperature data logger (Onset Computer Corporation, Bourne, MA, USA) was placed near each fish-rearing location, at a depth of approximately 0.5m below the water surface, which collected data at 15-minute intervals throughout the study. Water column chlorophyll samples were collected in 1-liter HDPE bottles, placed on ice and brought back to the laboratory for analysis. Chlorophyll samples were filtered upon return to the laboratory and the filters were frozen until further analysis. Water column nutrient samples were collected in 125 ml HDPE bottles, placed on ice, and then refrigerated when returned to the laboratory; constituents measured were total phosphorous, phosphate-phosphorous, ammonia-nitrogen, nitrate-nitrogen, total nitrogen, and dissolved organic carbon. Dissolved constituents were determined on samples following filtering through a 0.2 μm Millipore polycarbonate membrane filter; total concentrations were determined on non-filtered samples.

#### Food web

Juvenile Chinook salmon were reared in floating enclosures constructed of a rigid frame of 25.4 mm PVC pipe wrapped with an extruded plastic mesh with openings of 6.3 mm. The mesh size allowed for free movement of water, zooplankton and other invertebrates while keeping fish inside of enclosures. The plastic mesh was secured to the pvc frame with plastic zip ties. Enclosures were 1.2 m long x 1.2 m wide x 0.6 m deep. Enclosures were placed into each of the three study locations (agricultural floodplain wetland, perennial floodplain channel, and Sacramento River; Fig 1). Enclosures in the agricultural floodplain wetlands, where water depth did not fluctuate, were secured to metal posts driven into the ground with their top mesh even with the water surface. Floats were attached to the top of the cages in the perennial canal and Sacramento River, causing the cages to float with the top mesh directly at the water surface. These floating enclosures were attached to wood pilings via a tethered line that allowed the cages to float at the water’s surface as water elevations changed. The agricultural floodplain wetland location consisted of a 0.81 ha farm field that had a single inflow from a supply ditch and a single exit. Water depth was maintained at approximately 0.46 m by boards placed into an irrigation box for a total standing-water volume of approximately 3722 m^3^ in the field. The field, usually planted to rice, had been fallow the previous year when naturally recruited herbaceous vegetation had been allowed to grow. Previous studies have shown similar zooplankton composition in fallow fields and post-harvested rice fields at the study location [22]. The field was flooded using water from the adjacent irrigation canal on January 18, 2016, 32 days prior to the placement of the experimental fish.

Juvenile Chinook Salmon from the Feather River hatchery were transported to the study site via a fish transportation trailer tank where they were tagged with 8 mm passive integrated transponder (PIT) tags implanted into the abdominal cavity [23]. This study was carried out in strict accordance with the recommendations in the UC Davis Institutional Animal Care and Use Committee (Protocol # 18883). At the end of the experiment, fish were euthanized with a quick blow to the head as approved in our IACUC protocol and placed on ice and frozen for further tissue analysis.

Prior to planting into the enclosures, the hatchery fish were held for two days in large plastic tanks at the agricultural wetland site to ensure fish health and successful tag retention. Fish were scanned for PIT tag ID, measured, weighed, and placed into enclosures on February 19, 2016. Three enclosures were placed at each site, and 10 fish were placed into each enclosure. Fish were then sampled on days 9, 16, and 23 after initial planting. During sub-sampling, fish were removed from the enclosure, placed into a cooler, individually scanned, measured, weighed, and then placed back into the enclosure. Following the final measurement on March 11, 2016, fish were euthanized and immediately frozen for future gut content and stable isotope analysis for a concurrent project.

Zooplankton and macro-invertebrate samples were collected weekly at each site. To collect zooplankton, a 30 cm diameter 153 μm mesh net was used [22, 24]. The net was attached to a 5 m rope that was thrown into the water and retrieved while maintaining the entire net below the water surface. The net was thrown four times and all four throws were composited into a single sample. Following the fourth retrieval, the sample was rinsed from the net into the collecting cup that was then rinsed into a Whirl-Pak bag, preserved with 95% ethanol and stained with rose bengal. Samples were then taken back to the laboratory for enumeration and identification. Samples were rinsed through a 150 μm mesh and then emptied into a beaker. The beaker was then filled to a known volume to dilute the sample, depending on the density of individuals within the sample, and then sub-sampled with a 1 ml large bore pipette. If densities were still too great for enumeration, the sample was split using a Folsom splitter before sub-sampling with the pipette. The dilution volume, number of splits, and number of aliquots removed were recorded and used to obtain total estimates of invertebrates. Zooplankton samples were sorted until a minimum of 500 individuals were counted within a complete sub-sample. If less than 500 individuals were counted, another subsample was enumerated. Invertebrates were identified with the aid of a dissecting microscope at 4-times magnification to the lowest taxonomic level possible using keys [25–27]. Copepods were only identified to family. Terrestrial invertebrates were rare and not included in final counts. Benthic invertebrate sampling was not included in this study, as previous studies in these locations and many others within the Sacramento River system have found the vast majority of stomach contents of wild juvenile Chinook Salmon to be comprised of pelagic organisms [28].

#### Estimates of ecosystem productivity

Aquatic ecosystem metabolism is often expressed as net ecosystem productivity (NEP), which is determined as the difference between all photosynthetic energy produced in the system (gross primary productivity, GPP) and the sum of all energy used by organisms (ecosystem respiration, ER). We developed estimates of net ecosystem production in each habitat using two distinct approaches: ecosystem carbon budgeting, which indirectly estimates carbon flux via mass balances of physical inputs and outputs; and secondly, we constructed an aquatic metabolic model using inverse modeling in the StreamMetabolizer package (version 0.10.8) in R (version 3.3.2) [29].

To develop the mass balanced carbon budgets, we used an aquatic community production model to calculate biomass and production rates of primary producers (phytoplankton) as well as biomass and consumption rates of primary consumers (zooplankton) in each habitat (Table 1). Primary production biomass (PB) was calculated using an empirical model where the mass of carbon was calculated from observed chlorophyll-a abundance (32 mg C mg ^-1^ Chlorophyll-a) [30] and a production equation described in Lopez et al. [31] (Table 1). Zooplankton biomass (ZB) was calculated by obtaining dry weight from literature values. If the exact species could not be identified, an average dry weight of the published genera was used for the calculation. To determine the amount of carbon for each zooplankton taxa, a constant of 0.48 times dry weight was used as described in Andersen and Hessen [32]. Phytoplankton primary productivity (PP) and zooplankton grazing rate (ZG) was calculated using equations in Lopez et al. [31]. The ratio of the rates at which zooplankton graze organic carbon and the rate at which primary production converts inorganic carbon to organic via photosynthesis (ZG:PB) can be used to estimate if the autotrophic community is producing sufficient primary production to support the observed standing stock of zooplankton. A ratio less than 1 indicates that greater primary production is occurring within the habitat than is being consumed by zooplankton. Conversely, a ratio greater than 1 indicates a greater observed abundance of zooplankton than can be supported from primary production alone, thus inferring contribution from additional carbon sources. Due to unknown propagation of error through the model, no error is presented in the model results.

**Table 1:** Computation used for biomass, production and carbon flow. Computations and table adapted from Lopez, Cloern [31].

Index	Description	Units	Computation
PB	Plankton Biomass	mg C m^-3^	= 32(Chl *a*)
ZB	Zooplankton Biomass	mg C m^-3^	*= fz* DWi/1000
PP	Phytoplankton Primary Productivity	mg C m^-3^d^-1^	= (1/H) (0.85 Pg– 0.015 PB*H); Pg = 3.36(Chl a) (E/k)
ZG	Zooplankton Grazing Rate	mg C m^-3^d^-1^	= 0.95m_i_^0.8^ e^α (T–T')^ (1- e ^-0.01PB^)
ZB:PB	Potential Grazing Pressure	fraction	= ZB/PB
ZG:PB	Phytoplankton Biomass Grazed Daily	fraction d^-1^	= ZG/PB
ZG:ZB	Zooplankton Daily Ration	fraction d^-1^	= ZG/ZB
ZG:PP	Primary Production Grazed Daily	fraction	= ZG/PP

Chl a, chlorophyll a (mg m^-3^); ai, abundance of zooplankton taxon i (number m^-3^); mi, carbon biomass (μg) of zooplankton taxon i; fz, 0.48 from Andersen and Hessen (32); DWi, dry weight (μg) of zooplankton taxon i; Pg, areal gross primary productivity; H, mean water depth (m); E, daily surface irradiance (Einsteins m^-2^ d^-1^, PAR); k, attenuation coefficient (m^-1^); T, water temperature (°C).

Daily surface irradiance data were obtained from the nearby Davis station (station 6) at http://www.cimis.water.ca.gov. Daily surface irradiance was averaged throughout the study period at 138 watts per square meter and then converted to Einsteins (E) for calculations. Because no in situ measurements were collected for light attenuation within the water bodies, an attenuation coefficient of 1 was used for all three habitats throughout the study. This value represents an average of values from the literature and was used because extreme variability in turbidity values—due to frequently changing flow and wind conditions—complicated the use of periodically collected empirical data.

#### Modeling aquatic metabolism

Stream metabolism parameters—gross primary productivity (GPP), ecosystem respiration (ER) and net ecosystem productivity (NEP)—were modeled using the StreamMetabolizer package (version 0.10.8) in R (version 3.3.2) [29]. The hierarchical Bayesian model “bayes” within StreamMetabolizer estimates oxygen concentration at a 15-minute time step and models daily values of GPP, ER, and NEP. These parameters were then averaged and standard deviation calculated for the time period of the experiment. Model inputs included dissolved oxygen (mg/L), dissolved oxygen (percent saturation), depth (m), water temperature (degrees C), surface irradiance (μmol m^2^s^-1^), and discharge (m^3^s^-1^) from sources described previously. Data manipulation included using a spline interpolation to transform hourly surface irradiance data at http://www.cimis.water.ca.gov to 15-minute data. All interpolated values that were less than zero were replaced with a zero value.

#### Statistical analysis

For analysis of fish size at initial planting, the Shapiro Wilks test showed that the data were not normally distributed, so outliers were removed and a non-parametric Kruskal-Wallis test was used to determine variation between treatment groups at the start of the experiments. To determine differences in growth rates between habitats over time, statistical analysis was conducted in JMP Pro version 12.0.1. Because the fish were individually marked and measured several times throughout the experiment, a mixed model repeated measures analysis was used to determine differences in growth between study habitats. Time and location were the fixed effects in the model used. Only fish that were sampled throughout the study were included into the statistical analysis.

## Results

### Hydrology

**Fig 2:**
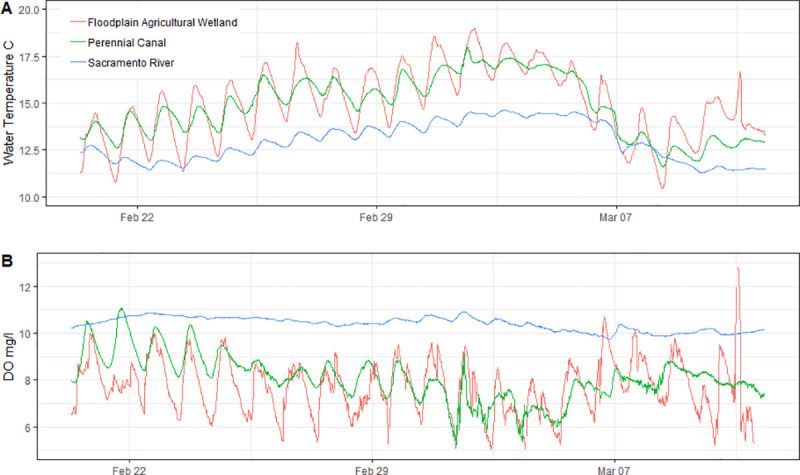
Water Temperature (degrees Celsius) (A) and Dissolved Oxygen (DO) in mg/l (B) continuously sampled every 15 minutes at flooded floodplain agricultural wetland, perennial drainage canal and Sacramento River habitats.

There was no measurable velocity across the floodplain agricultural wetland, and discharges into and out of the field ranged from zero to 0.02 m^3^s^-1^ throughout the study. Discharge in the floodplain perennial drainage canal ranged from 0.82 m^3^s^-1^ to 68.0 m^3^s^-1^, while discharge in the Sacramento River ranged from 314.3 m^3^s^-1^ to 1,427.2 m^3^s^-1^. Stage in the floodplain agricultural wetland was stable throughout the study, while stage varied by 2.75 m in the canal and 5.00 m in the Sacramento River. Mean water residence times calculated for the floodplain agricultural wetland, perennial drainage canal and Sacramento River were 2.15 days, 23.5 seconds, and 1.7 seconds, respectively. Water temperature was most variable on the floodplain agricultural wetland habitat with the highest highs and the lowest lows, and the Sacramento River habitat was the most stable with little fluctuation throughout the study (Fig 2, Table 2). Similar to temperature, dissolved oxygen was highly variable in the floodplain agricultural wetland habitat and very stable in the Sacramento River (Fig 2). Dissolved oxygen in the floodplain agricultural wetland was below saturation throughout most of the study with the exception of days when wind mixed the shallow water. Dissolved oxygen in the perennial drainage canal had a similar pattern to that of the floodplain agricultural wetland, but with smaller daily fluctuations and a higher average dissolved oxygen concentration.

**Table 2:** Mean values of water quality data collected from the floodplain agricultural wetland, perennial canal, and Sacramento River study sites.

Water Quality Constituent	Floodplain agricultural wetland	Perennial canal	Sacramento River
**Average Temperature (°C)**	15.22	15.28	13.21
**Maximum Temperature (°C)**	19.00	18.02	14.64
**Minimum Temperature (°C)**	10.78	12.60	11.44
**Average Dissolved Oxygen (mg l**^**-1**^**)**	7.67	8.11	10.46
**Maximum Dissolved Oxygen (mg l**^**-1**^**)**	10.71	11.07	10.91
**Minimum Dissolved Oxygen (mg l**^**-1**^**)**	4.86	5.07	9.72
**Average Total Phosphorous (mg l**^**-1**^**)**	0.21	0.90	0.11
**Average Phosphate-Phosphorus (mg l**^**-1**^**)**	0.14	0.29	0.02
**Average Nitrate-Nitrogen (mg l**^**-1**^**)**	<0.01	1.41	0.23
**Average Ammonium-Nitrogen (mg l**^**-1**^**)**	0.12	0.05	0.01
**Dissolved Organic Carbon (mg l**^**-1**^**)**	12.00	9.63	2.87

### Water chemistry

**Fig 3:**
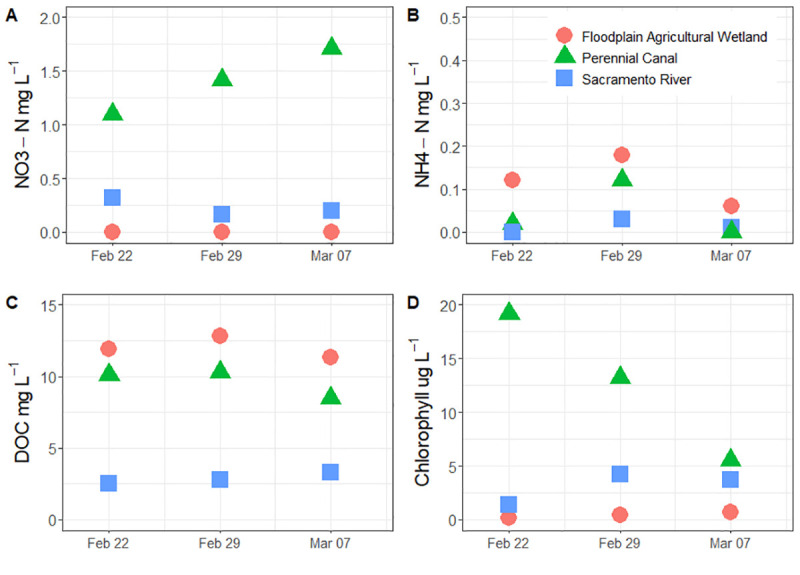
Nitrate-nitrogen (NO_3_-N) mg l^-1^ (A), Ammonium-nitrogen (NH_4_-N) mg l^-1^ (B), Dissolved organic carbon (DOC) mg l^-1^ (C), Chlorophyll-a μg l^-1^ (D) sampled at flooded floodplain agricultural wetlands, perennial canal, and Sacramento River habitats. Note that y-axis scales are variable between graphs so that differences in habitats may be distinguished.

Nitrate-nitrogen values were highest in the perennial drainage canal throughout the study and ranged from 1.10 to 1.71 mg l^-1^ (Fig 3). Sacramento River nitrate-nitrogen values were relatively stable and ranged from 0.16 to 0.32 mg l^-1^. Nitrate-nitrogen values were non-detectible (<0.01 mg l^-1^) for all samples collected in the floodplain agricultural wetland throughout the study. Ammonium values were relatively low across all of the habitats sampled, with the highest values being in the floodplain agricultural wetland habitat, ranging from 0.06 to 0.18 mg l^-1^ (Fig 3). Dissolved organic carbon (DOC) was highest in the floodplain agricultural wetlands, which ranged from 11.3 to 12.8 mg l^-1^ (Fig 3). The Sacramento River had the lowest DOC values, which ranged from 2.5 to 3.3 mg l^-1^.

Chlorophyll values were highest in the perennial drainage canal and lowest in the floodplain agricultural wetland (Fig 3). Chlorophyll declined throughout the study in the perennial drainage canal from a high of 19.2 μg l^**-1**^ to a low of 5.5 μg l^-**1**^. Sacramento River chlororphyll values increased from the initial sampling of 1.4 μg l^**-1**^ to 3.7 μg l^**-1**^ during the final sample. In the floodplain agricultural wetland, chlorophyll values varied little (0.2 to 0.7 μg l^**-1**^) and remained relatively small compared to the other two locations.

### Ecosystem budgeting: Zooplankton and phytoplankton biomass and vital rates

**Fig 4:**
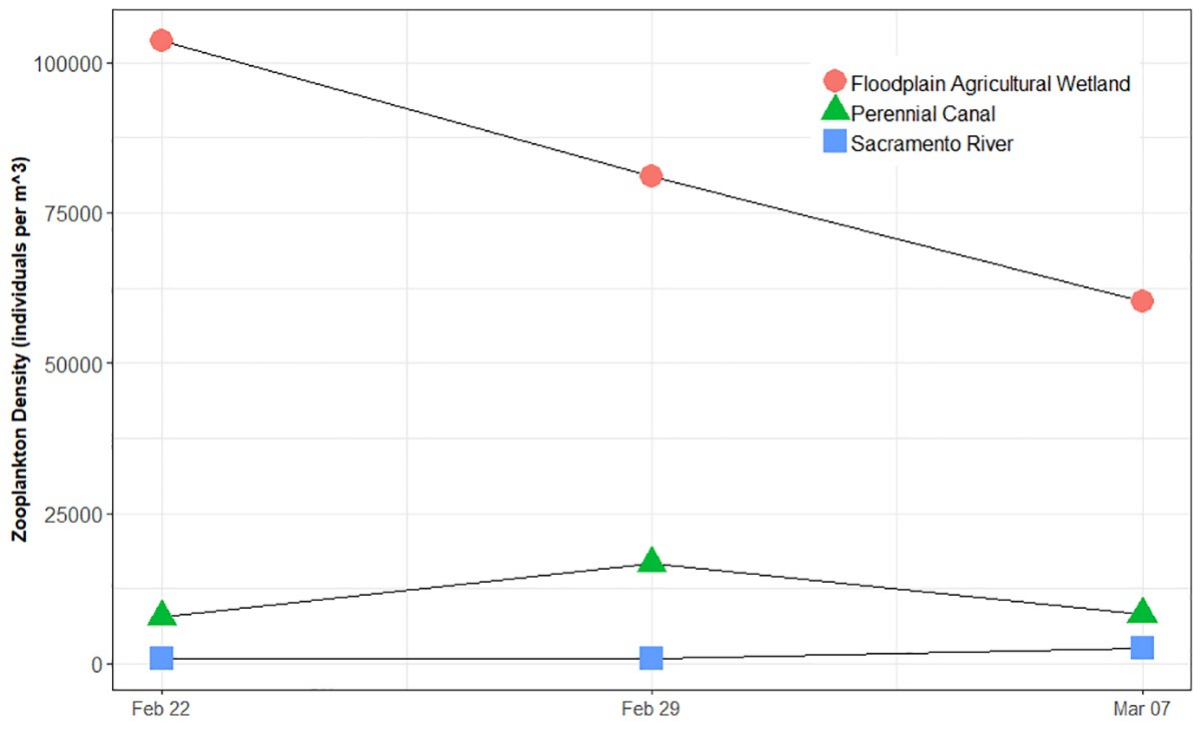
Estimated zooplankton density (individuals/m^3^) in the floodplain agricultural wetland, perennial canal, and Sacramento River habitats.

A total of 18, 20, and 15 taxa were identified from the floodplain agricultural wetland, perennial drainage canal and Sacramento River, respectively, throughout the study (Table 3). Mean zooplankton density for the three sample periods was 81,031+/- 21,702 sd individuals/m^3^ in the floodplain agricultural wetlands; 11,831 +/- 4946 sd individuals/m^3^ in the perennial canal; and 1,529 +/- 1023 sd individuals/m^3^ in the Sacramento River. Numbers of zooplankton in the floodplain agricultural wetland declined from a high of 103,714 individuals m^-3^ to a low of 60,321 individuals m^-3^ throughout the study, yet remained much higher than either the perennial drainage canal or the Sacramento River habitats (Fig 4).

**Table 3:** Taxa, mean density (standard deviation), and proportion of zooplankton collected during three sampling periods from the floodplain agricultural wetland, perennial canal, and Sacramento River habitats. Samples were collected adjacent to the fish enclosures.

Floodplain agricultural wetland			Perennial canal			Sacramento River		
Taxa	Density m^3^	Proportion	Taxa	Density m^3^	Proportion	Taxa	Density m^3^	Proportion
D. pulex	21,111.06 (12,658.17)	0.31	Chydorus	6090.65 (4423.34)	0.52	Bosmina	467.42 (620.97)	0.26
Ceriodaphnia	9025.50 (5042.65)	0.13	Cyclopidae	1671.38 (1498.12)	0.14	Cyclopidae	322.94 (344.98)	0.18
Bosmina	6212.85 (4914.60)	0.09	D. pulex	864.02 (482.63)	0.07	Chydorus	198.30 (198.30)	0.11
Chydorus	6041.84 (4293.50)	0.09	Bosmina	845.13 (1292.16)	0.07	Rotifera	169.97 (157.72)	0.09
Eucypris	5526.66 (4763.37)	0.08	Ceriodaphnia	493.38 (258.51)	0.04	Acanthocyclops	99.15 (20.03)	0.06
Cyclopidae	3991.78 (4709.75)	0.06	C. alona	332.86 (255.25)	0.03	D. pulex	99.15 (100.15)	0.06
S. mixtus	3990.30 (2163.54)	0.06	Rotifera	276.20 (229.59)	0.02	Calinoida	84.98 (0.00)	0.05
D. laevis	2769.71 (2920.25)	0.04	Collembola	254.95 (280.44)	0.02	Ceriodaphnia	84.98 (0.00)	0.05
Acanthocyclops	2136.58 (1413.17)	0.03	Acanthocyclops	212.46 (135.12)	0.02	Harpacticoid	56.65 (0.00)	0.03
Rotifera	1186.26 (2370.81)	0.02	Chironomidae	141.64 (120.19)	0.01	Nematoda	56.65 (0.00)	0.03
Calinoida	1077.73 (1748.52)	0.02	S. mixtus	113.31 (40.06)	0.01	Chironomidae	37.77 (16.35)	0.02
Daphniidae	970.25 (1002.85)	0.01				C. alona	28.32 (0.00)	0.02
Eurycercus	637.39 (0.00)	0.01				Ceratopogonidae	28.32 (0.00)	0.02
C. alona	542.71 (461.46)	0.01				Megaloptera	28.32 (0.00)	0.02
Chironomidae	361.26 (241.13)	0.01				Tardigrades	28.32 (0.00)	0.02

**Fig 5:**
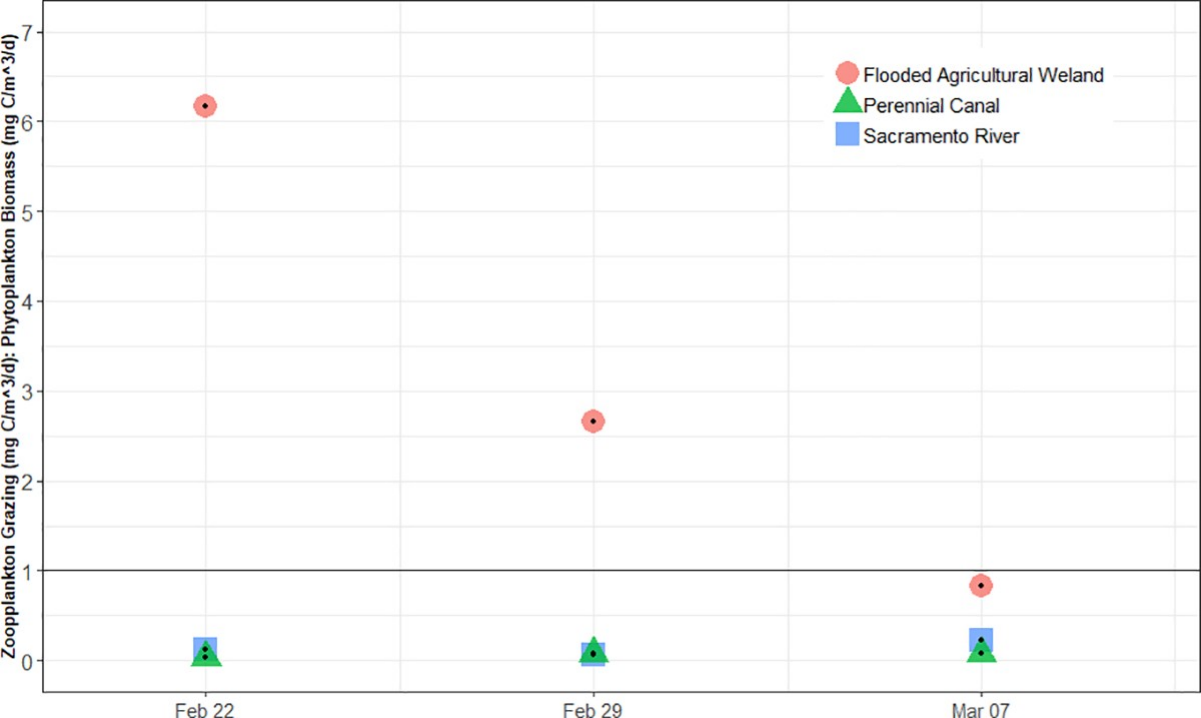
Ratio of zooplankton grazing rate (mg C m^-3^d^-1^) to phytoplankton production rate (mg C m^-3^d^-1^) in floodplain agricultural wetlands, perennial canal, and Sacramento River. A value greater than 1 indicates more grazing than can be supported by the standing biomass of phytoplankton, thus indicating an alternate source of carbon is fueling community metabolism within the habitat in question.

Zooplankton grazing to phytoplankton biomass (ZG:PB) ratios varied over time and between habitats. The floodplain agricultural wetland ratio was the highest throughout the study but declined from a high of 6.67 to a low of 0.96 (Fig 5). Both the perennial drainage canal (range of 0.03 to 0.08) and Sacramento River (range of 0.06 to 0.22) locations remained well below a ratio of 1.

### Aquatic metabolism modeling results

**Fig 6:**
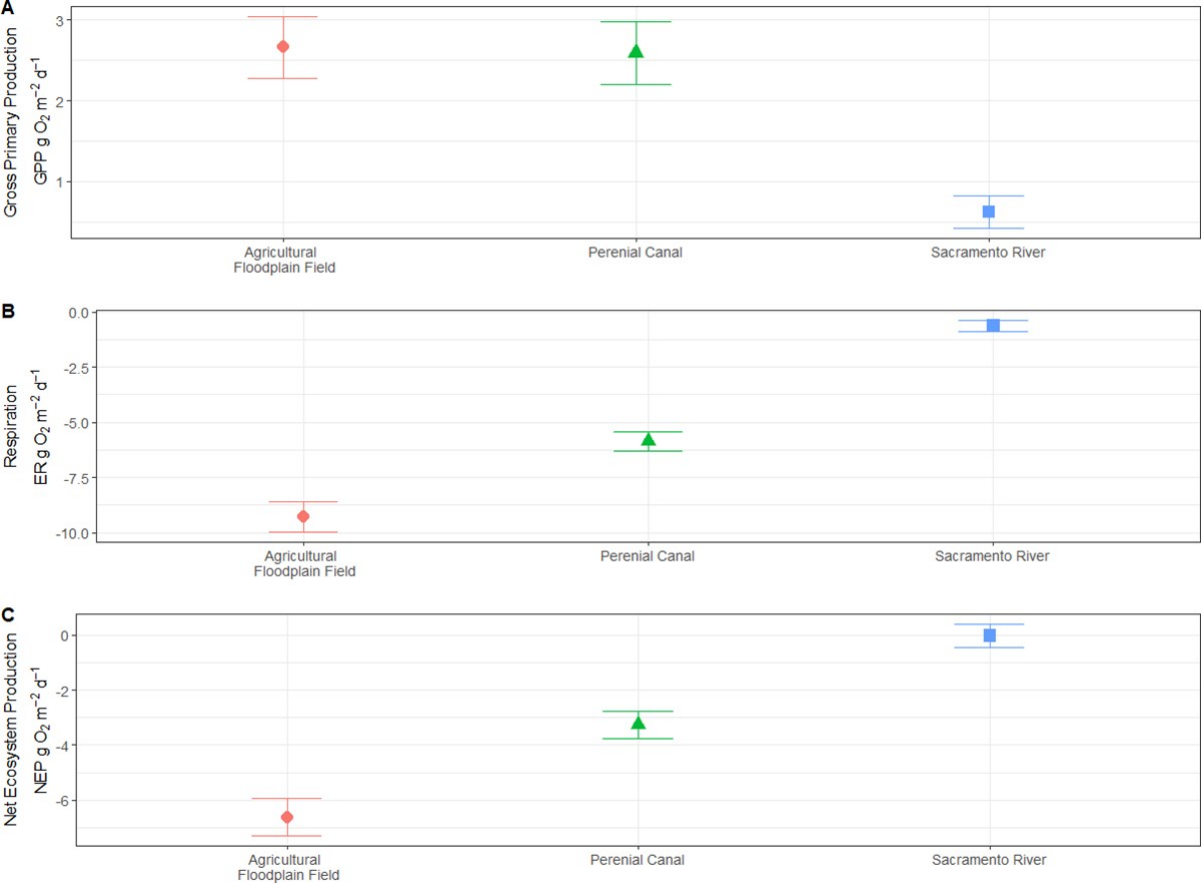
Model output of A) gross primary productivity (GPP, g O_2_ m^-2^d^-1^), B) ecosystem respiration (g O_2_ m^-2^d^-1^), and C) net ecosystem productivity (NEP, g O_2_ m^-2^d^-1^).

**Fig 7:**
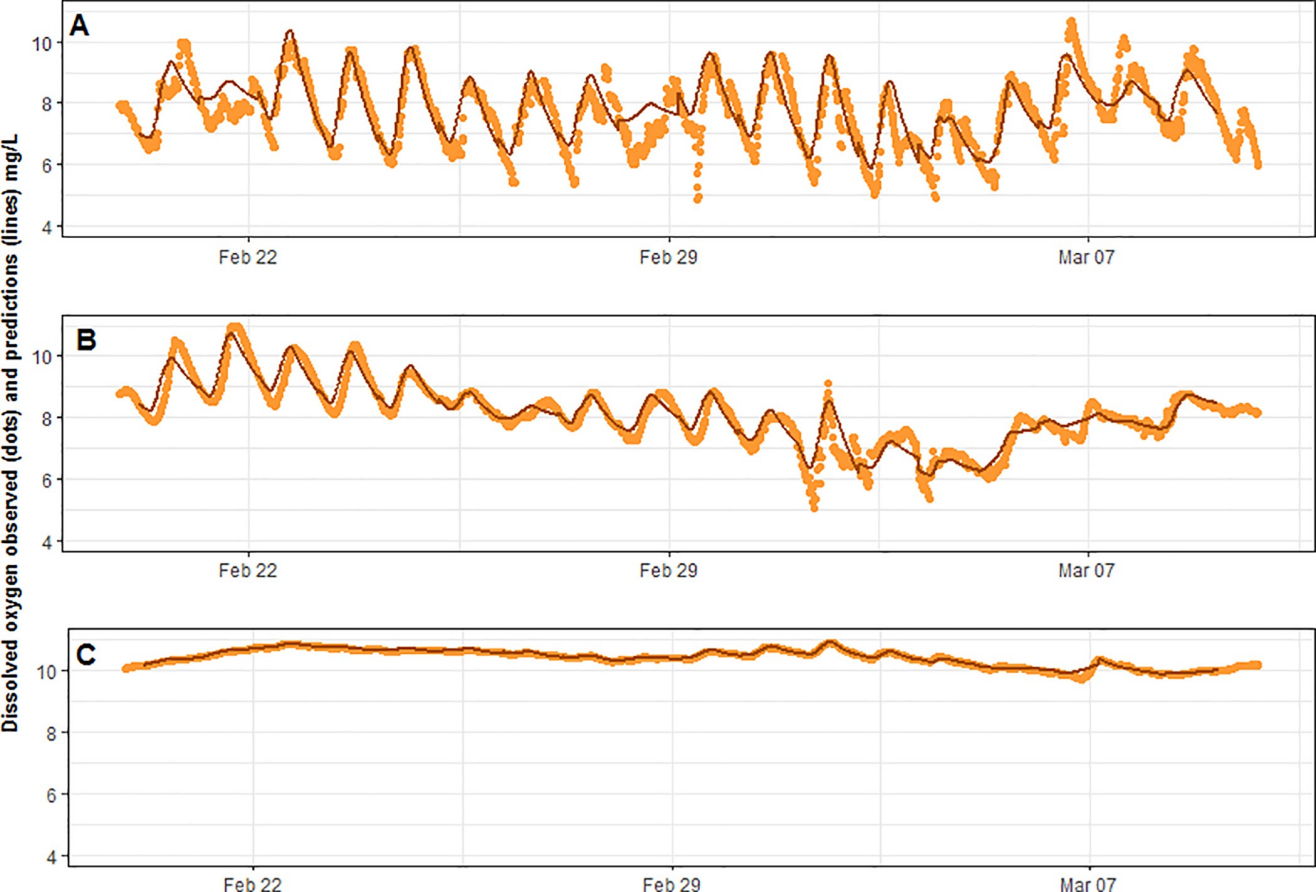
Observed and modeled Dissolved Oxygen data for A) Floodplain agricultural wetland, B) Perennial canal, and C) Sacramento River output from Streammetabolizer package in R.

Modeled estimates for both the floodplain agricultural wetland and perennial drainage canal had similar daily average gross primary production (GPP) values of 2.66 (+/- 1.51 sd) *g* O_2_ *m*^*-2*^*d*^*-1*^ and 2.59 (+/- 1.53 sd) *g* O_2_ *m*^*-2*^*d*^*-1*^ respectively, while the Sacramento River was much lower 0.62 (+/- 0.79 sd) *g* O_2_ *m*^*-2*^*d*^*-1*^ (Fig 6). Ecosystem respiration (ER) values varied greatly between the three habitat types, with the floodplain being the most negative at -9.33 (+/- 2.69 sd) *g* O_2_ *m*^*-2*^*d*^*-1*^ (more respiration) and the Sacramento River site being the least negative -0.64 (+/- 0.98 sd) *g* O_2_ *m*^*-2*^*d*^*-1*^ (less respiration). When respiration is subtracted from gross primary production, the result is net ecosystem production (NEP). NEP values were estimated to be -6.66 (+/- 2.65 sd) *g* O_2_ *m*^*-2*^*d*^*-1*^, -3.27 (+/- 2.05 sd) *g* O_2_ *m*^*-2*^*d*^*-1*^, and -0.02 (+/- 1.66 sd) *g* O_2_ *m*^*-2*^*d*^*-1*^ in the floodplain agricultural wetland, perennial drainage canal and Sacramento River respectively (Fig 6). The stream metabolism model had a difficult time with the variability in dissolved oxygen in the floodplain agricultural wetland. The modeled results failed to reach the minimum dissolved oxygen values observed in the agricultural wetlands. This likely resulted in an underestimation of the ER and as a result increased the NEP output values (Fig 7).

### Fish growth

**Fig 8:**
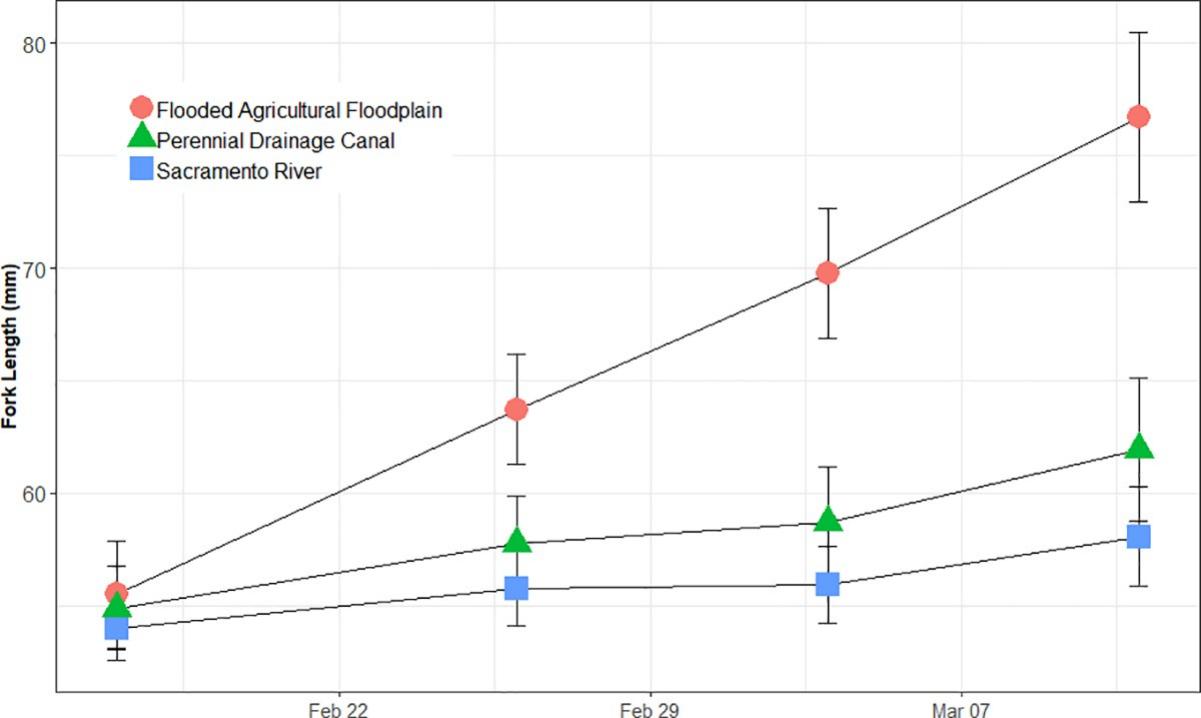
Fork length of juvenile fish reared in a flooded floodplain agricultural wetland, perennial drainage canal, and Sacramento River. Circles are habitat mean fork length, and error bars are standard deviation.

**Fig 9:**
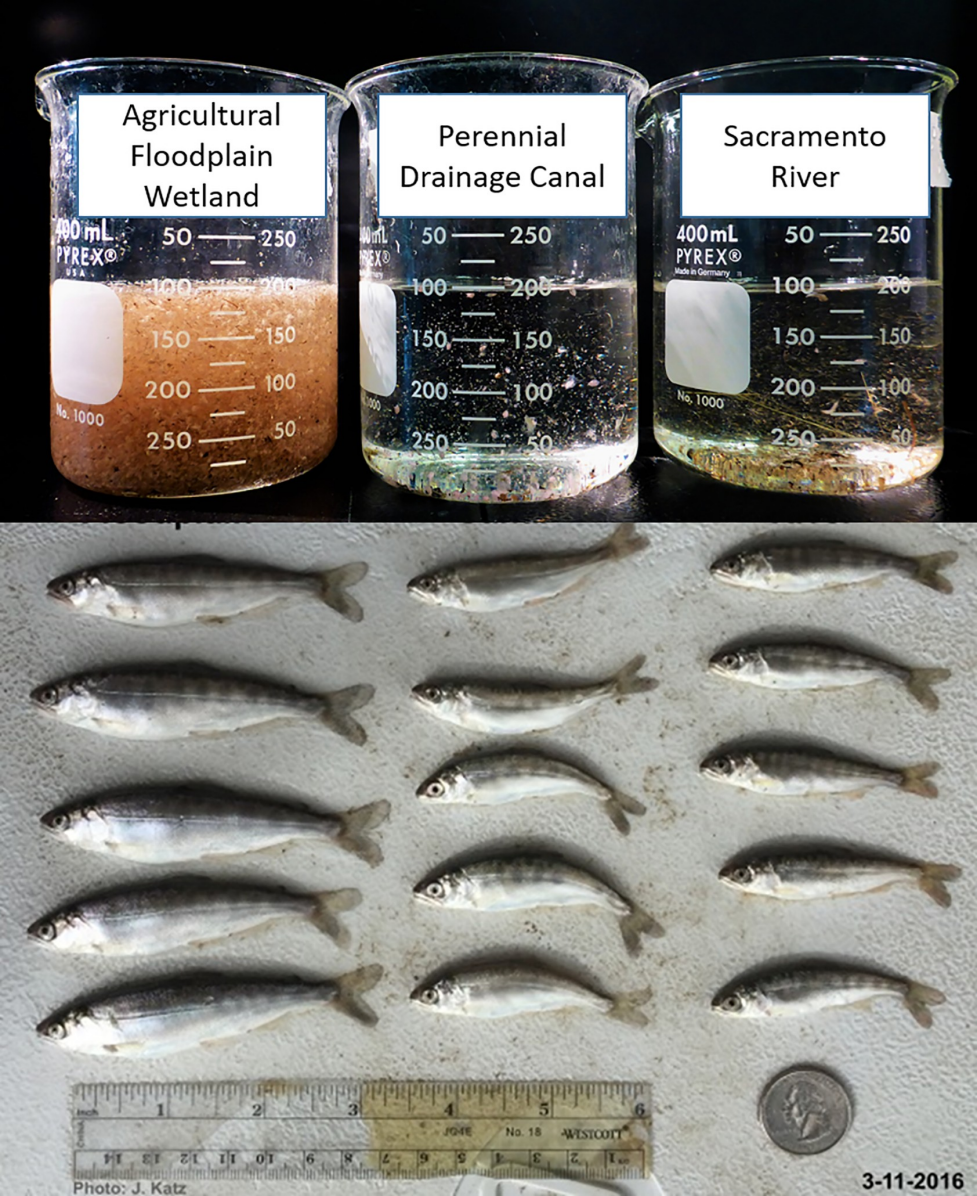
Photo of zooplankton (March 25, 2016) sample and representative fish (March 11, 2016) from each of the three habitat types: Flooded agricultural wetland, perennial drainage canal, and Sacramento River. Note the greater abundance of zooplankton and larger associated size of fish from the flooded agricultural floodplain habitat compared to the two other locations.

Juvenile Chinook Salmon averaged 54.8 mm (+/- 2.00 sd) across all habitats when initially placed into the enclosures (Fig 9). Throughout the study, fish in the floodplain agricultural wetland, perennial canal, and Sacramento River grew in fork length, on average 0.93 (+/-0.15 sd) mm d^-1^; 0.31 (+/- 0.10 sd) mm d^-1^; and 0.18 (+/- 0.09 sd) mm d^-1^, respectively. At the conclusion of the study, the average fork lengths were floodplain agricultural wetland = 76.7 mm (+/- 3.78 sd); perennial drainage canal = 62.0 mm (+/- 3.17 sd); and Sacramento River = 58.2 mm (+/- 2.45 sd) (Fig 8). Fish in the floodplain agricultural wetlands were significantly longer in fork length than the fish in either the perennial drainage canal or the Sacramento River (p < 0.0001) and the perennial drainage canal fish were significantly longer in fork length that the fish in the Sacramento River (p<0.0001) (Fig 9).

In the floodplain agricultural wetland habitat, six fish escaped through a hole in one of the enclosures shortly after initial stocking. Just prior to the final sampling event, another fish escaped and one died, leaving a total of 22 fish measured through the entire study. Of the seven escaped fish, five were recaptured after completion of the study during the draining of the agricultural wetland. In the Sacramento River habitat, one fish died during removal from the enclosure for measurement, for a total of 29 fish that were measured through the study duration. All 30 fish initially planted in the perennial drainage canal habitat were present on the final day of the study.

## Discussion

The purpose of our study was to examine food web responses across a range of habitat conditions in a river-floodplain complex. Like many large-river systems, the Sacramento River and its adjacent floodplain (Yolo Bypass) have been heavily modified for flood management and agriculture [7]. For example, levees and weirs have substantially reduced the connectivity between the Sacramento River and the Yolo Bypass, reducing the frequency and duration of seasonal inundation. Nonetheless, our study suggests that even heavily managed floodplains such as Yolo Bypass retain habitat attributes that enhance the productivity and diversity of the food web. Overall, we found support for our hypotheses that off-channel agricultural wetland habitat generates higher densities of zooplankton and increased growth rates of juvenile salmon as compared to adjacent perennial canal and river channels. While our study design relied on the use of caged hatchery fish and a heavily managed agricultural wetland as part of the habitat comparisons, these results are consistent with observations of zooplankton densities and wild fish growth rates during more natural uncontrolled flood events [18, 33, 34]. Below we describe some of the habitat attributes that may be responsible for these differences.

Fish in the inundated floodplain agricultural wetland grew significantly faster than fish in either the perennial drainage canal or the Sacramento River. Food resources in the Sacramento River were generally sparse compared to those on the floodplain, corroborating and informing past studies that have shown fish reared in river channels to have considerably slower growth rates, compared to those reared in adjacent off-channel habitats [11, 19, 35]. These results supported our hypotheses that inundated floodplain agricultural wetland habitats should exhibit: 1) greater heterotrophic metabolic activity, 2) higher densities of zooplankton, and 3) rapid growth rates of juvenile salmon, as compared to adjacent perennial canal and river channel habitats. Empirical support for the second and third hypotheses was dramatic: zooplankton densities on the floodplain agricultural wetland were 53 times more abundant, on average, than in the river and nearly 7 times more abundant, on average, than in the canal, while juvenile Chinook Salmon raised on the floodplain agricultural wetland grew at 0.92 mm/day, a rate 5 times faster than fish raised in the adjacent river habitat (0.18 mm/day) and 3 times faster than fish raised in the canal habitat (0.31 mm/day).

Both modeling approaches returned results supporting an elevated contribution by heterotrophic pathways to the food web of the floodplain agricultural wetland habitat compared to those of the perennial canal and river channel habitats. First, the rates of primary production on the floodplain estimated by mass balance were generally insufficient to support the estimated grazing pressure from the observed biomass of zooplankton. Zooplankton grazing to phytoplankton biomass ratios (ZG:PB) greater than 1 indicate more grazing from zooplankton than can be supported by the standing biomass of phytoplankton, thus signifying an alternate carbon source is contributing to metabolic activity within the community. Recorded ZG:PB ratios from the floodplain were high as 6.67, indicating dramatically higher rates of grazing than could be supported by estimated phytoplankton biomass alone. Meanwhile, ZG:PB values in the perennial drainage canal (0.03 to 0.08) and Sacramento River (0.06 to 0.22) remained well below 1 for the duration of the study indicating that in these habitats autotrophic production alone was theoretically capable of supporting estimated grazing pressures. It is possible that high turnover of autotrophic producers could partially account for high zooplankton biomass, but more complex modeling than mass balance is necessary to understand this flux. While these results do not provide specific estimates of the contributions of heterotrophic relative to autotrophic production within each habitat, they do imply that heterotrophic production plays a much larger role in fueling the pelagic food web in the ephemeral floodplain than in either of the perennial channel habitats studied.

Additionally, community respiration far exceeded oxygen production from photosynthesis on the floodplain, as evidenced by the modeled rate of oxygen consumption (ER, -9.33 (+/- 2.69 sd) *g* O_2_ *m*^*-2*^*d*^*-1*^) versus the rate at which the model estimated oxygen to be produced by primary production (GPP, 2.66 (+/- 1.51 sd) *g* O_2_ *m*^*-2*^*d*^*-1*^) (Fig 7). Primary production is generally regulated by either bottom-up limitations (nutrients) or top-down grazing pressure from primary consumers, in this case zooplankton [36, 37]. The floodplain habitat contained high densities of zooplankton, yet exhibited low concentrations of chlorophyll. While the higher zooplankton densities seen on the floodplain compared to adjacent channels is consistent with earlier studies [18, 34], the chlorophyll levels documented in the agricultural wetland were much lower than those observed by previous studies during flood events on Yolo Bypass [34]. We found that the floodplain contained high densities of zooplankton, yet exhibited relatively low concentrations of chlorophyll. This apparent paradox is easily explained if high primary production rates are kept in check by particularly intense grazing pressure generated by unexpectedly abundant zooplankton sustained at high abundance by alternate heterotrophic energy sources. Such conditions would result in low observed pelagic chlorophyll biomass, high observed zooplankton biomass, and high modeled GPP, just as we found in the managed floodplain habitat of this study. Benthic algae could also be contributing to high modeled GPP values and not accounted for in the water column chlorophyll values. This unaccounted source of primary production is likely important and should be considered for future work. We therefore suggest that the floodplain habitat’s highly productive community metabolism (characterized by relatively high GPP, high ZB, low PB) is likely being fueled by high rates of both heterotrophic and autotrophic production.

This finding runs counter to a commonly held conceptual model among many in the scientific and management community that focuses on autotrophic production as the base of aquatic food webs [38]. The misperception that chlorophyll, pelagic chlorophyll in particular, is the primary measure of aquatic ecosystem productivity may become entrenched by the relative ease of measuring chlorophyll in the water column while it is inherently difficult to obtain a direct measure of the heterotrophic production, as we attempted in this study. In addition, benthic algal production as well as the benthic-pelagic interface—which is the primary nexus for the transfer of detrital production into the pelagic food web—are critical and understudied components of floodplain ecosystems. Our results suggest that observed water column chlorophyll abundances, considered in isolation, are in fact a highly unreliable proxy for an aquatic food web’s productive capacity or potential.

Ephemerally abundant floodplain food resources may be utilized by migratory fish that gain access to hydrologically activated floodplain habitats. Floodplain-derived food web resources may also be exported to downstream water bodies as the floodplains drain, thereby subsidizing in-river food webs and making floodplain-derived food web resources available to fish populations confined to downstream river channels. While this study design relied on the use of caged hatchery fish and on intensively managed agricultural wetlands, these results are consistent with observations of zooplankton densities and wild fish growth rates documented during more natural uncontrolled flood events [18, 33, 34].

Inundation of floodplains facilitates the decomposition of terrestrial vegetation, allowing floodplain soils to leach labile carbon into the water column. These detrital carbon sources, as well as methane produced in the oxygen-poor wetland soils, can support the single-celled organisms and methane-oxidizing bacteria [12] that are the base of heterotrophic pathways capable of fueling highly productive food webs, even in the absence of significant phytoplankton production [14]. In shallow floodplain lakes in the Amazon River, for example, up to 84% of the amino acids in fish were derived from methane-oxidizing bacteria [12]. Data collected for a concurrent project from the stomach contents (primarily *D*. *pulex*) of fish used in this study found δ^13^C values generally more depleted than -35‰ (Tilcock et al. IN REVIEW). This isotopic evidence also supports the importance of heterotrophic food webs in floodplain enviroments, as it suggests that zooplankton consumed by floodplain-reared fish in this study had fed on bacteria exploiting terrestrial carbon leachate or methane produced in oxygen-poor floodplain soils [12, 39]. Other work on the Yolo Bypass has found that a detritivorous chironomid midge was an important food source for juvenile salmonids and other fishes utilizing the flooded Yolo Bypass during flood events [17, 18].

Below we discuss some of the hydrologic and biophysical habitat attributes that may also contribute to observed differences in stream metabolism across habitats, and we consider various implications for river management and conservation action. Differences in the abundance and availability of basal carbon resources were likely the largest contributing factors to observed differences in zooplankton abundance and juvenile salmon growth between the habitats, with temperature and water residence time also playing important contributing roles. During this study, in the Sacramento River we observed little productivity in general, either autotrophic or heterotrophic, as indicated by low zooplankton abundance and small diurnal variation in dissolved oxygen. In contrast the floodplain was a metabolic engine of both heterotrophic and autotrophic production, resulting in a standing stock of pelagic zooplankton, the primary forage of juvenile salmon, 53x greater than that observed in the starved food web of the adjacent channelized river.

Longer water residence times likely contribute to higher zooplankton densities by increasing DOC and labile carbon availability for microbial decomposition [40, 41]. Water residence times on the floodplain were more than 6 orders of magnitude greater than those in the river, likely contributing to the vastly more productive food webs observed in the slow, shallow waters of the off-channel habitat. Water temperatures in the shallowly inundated floodplain agricultural wetland and the perennial drainage canal were also warmer and more variable compared to those in the Sacramento River (Fig 2), a finding consistent with other studies in this region [18]. When water temperatures are higher, yet within physiological tolerances, and food resources are abundant, as documented on the floodplain, juvenile salmon growth rates can exceed those of fish in cooler habitats [42, 43]. Warmer temperatures also allow for increased enzymatic activity corresponding with increased rates of both microbial and algal production, resulting in more basal food web production [44]. Warmer temperatures observed on the floodplain relative to the river, therefore, likely support both increased fish activity as well as elevated densities of energy-dense zooplankton readily available within the water column. Abundant food and elevated activity rates synergistically combine to facilitate increased foraging efficiencies (caloric intake) and fuel the rapid juvenile salmon growth observed in floodplain habitats. While water temperatures were similar between the floodplain and the perennial canal, higher zooplankton densities on the floodplain likely supported higher foraging efficiencies, contributing to juvenile salmon growth rates 3x higher in the floodplain than in the canal (Fig 9).

Our results suggest that heterotrophic pathways are a major contributor to highly productive aquatic food webs in the floodplain habitats that are widely accepted as being critically important for rearing juvenile salmon [11, 18]. These results also make clear that different habitats—even those that are relatively close together and may be intermittently hydrologically connected—can support dramatically different food webs and may ultimately provide very different growth potential for juvenile salmon. This study also suggests that when “re-activated,” even highly altered and intensely managed floodplains such as farm fields in Yolo Bypass can retain habitat attributes that enhance the productivity and diversity of the aquatic food web, compared to the main-stem river. The relative importance of floodplain-derived food resources to the juvenile life stages of the salmonid life cycle suggests that habitat conditions which foster detrital food webs may deserve greater scrutiny in temperate river systems as important engines for the production of aquatic biomass, especially fish.

Hydrologic conditions typifying the ephemeral floodplain—shallower depths, warmer water, longer water residence times and greater availability of detrital carbon sources compared to deeper, colder, swifter water and primarily algal-based carbon sources in the adjacent river channel—appear to facilitate the dramatically higher rates of food web production observed in floodplain versus river channel habitats. These results suggest that hydrologic patterns associated with wet season flooding in Mediterranean-climate river systems can provide access to detrital carbon that is an important energy source for the production of fisheries resources and biomass. This study also demonstrates that mimicking natural hydrologic processes in agricultural valleys can help restore ecological function to highly altered temperate river ecosystems, even in intensively managed landscapes such as California’s Sacramento Valley. It is our hope that this study will inform conservation, farm, water, and fisheries professionals to better support landscape management actions that restore or approximate the natural long-duration flood patterns that facilitate the transfer of floodplain-derived resources into river food webs, where they are needed to support robust fisheries and to promote the recovery of imperiled fish species.
